# The Role of miRNAs in the Regulation of Endometrial Cancer Invasiveness and Metastasis—A Systematic Review

**DOI:** 10.3390/cancers13143393

**Published:** 2021-07-06

**Authors:** Klaudia Klicka, Tomasz M. Grzywa, Alicja Klinke, Aleksandra Mielniczuk, Paweł K. Włodarski

**Affiliations:** 1 Department of Methodology, Medical University of Warsaw, 02-091 Warsaw, Poland; klaudia.klicka@wum.edu.pl (K.K.); tomasz.grzywa@wum.edu.pl (T.M.G.); s073727@student.wum.edu.pl (A.K.); s073849@student.wum.edu.pl (A.M.); 2 Doctoral School, Medical University of Warsaw, 02-091 Warsaw, Poland; 3 Department of Immunology, Medical University of Warsaw, 02-097 Warsaw, Poland

**Keywords:** endometrial cancer, miRNAs, metastasis, invasiveness, migration, biomarker

## Abstract

**Simple Summary:**

Endometrial cancer (EC) is one of the most frequent cancers with increasing annual death rates. Therefore, it is of great clinical importance to understand the mechanisms of endometrial cancer invasiveness and metastasis. MiRNAs are small single-stranded RNAs that regulate gene expression. They were discovered to play a role in all steps of cancer development. This study aimed at conducting a systematic review of the role of miRNAs in endometrial cancer invasiveness and metastasis. The collected data demonstrate that miRNAs regulate EC invasiveness and metastasis by different targets. MiRNAs seem to be potential candidates for diagnostic and prognostic biomarkers, as well as possible therapeutic targets.

**Abstract:**

Endometrial cancer (EC) is the most common genital cancer in women with increasing death rates. MiRNAs are short non-coding RNAs that regulate gene expression on the post-transcriptional levels. Multiple studies demonstrated a fundamental role of miRNAs in the regulation of carcinogenesis. This systematic review is a comprehensive overview of the role of miRNAs in the regulation of cancer cell invasiveness and metastasis in EC. The literature was searched for studies investigating the role of miRNAs in the regulation of invasiveness and metastasis in EC. We explored PubMed, Embase, and Scopus using the following keywords: miRNA, metastasis, invasiveness, endometrial cancer. Data were collected from 163 articles that described the expression and role of 106 miRNAs in the regulation of EC invasiveness and metastasis out of which 63 were tumor suppressor miRNAs, and 38 were oncomiRNAs. Five miRNAs had a discordant role in different studies. Moreover, we identified 66 miRNAs whose expression in tumor tissue or concentration in serum correlated with at least one clinical parameter. These findings suggest a crucial role of miRNAs in the regulation of EC invasiveness and metastasis and present them as potential prognostic factors for patients with EC.

## 1. Introduction

**Figure 1:**
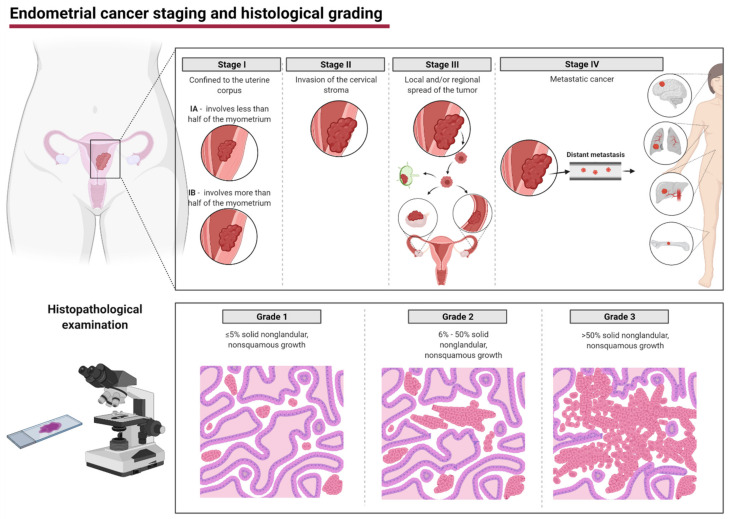
Federation of Gynecology and Obstetrics (FIGO) staging and histological grading of endometrial cancer. Figure was created using Biorender.com.

Endometrial cancer (EC) accounts for the most frequent cancers with growing incidence rates [1,2]. The outcome depends on the progression of the disease and applied treatment [3,4]. That makes effective management of EC risk factors, early diagnosis, and effective therapy strategies of EC the clinically important challenges. There are two most common EC staging classifications, TNM and The International Federation of Gynecology and Obstetrics (FIGO) that differentiate endometrial cancer tumors according to the depth of invasion and presence of metastases (Figure 1) [5]. Furthermore, there is a FIGO grading based on the level of glandular differentiation. A higher grade is associated with a non-glandular, non-squamous growth [6].

MiRNAs are small non-coding single-stranded molecules that regulate all hallmarks of cancer by influencing gene expression post-transcriptionally. The formation of miRNAs begins in the nucleus where polymerase II (Pol II) transcribes pri-miRNA. The pri-miRNA is cropped by the DROSHA complex and exported by exportin 5 to the cytoplasm where mature single-stranded miRNAs arise with the participation of DICER and Argonaute 2 (AGO2) [7,8]. During carcinogenesis, the profile of miRNAs expression undergoes a substantial dysregulation [9,10]. It is a result of multiple changes, including amplification and deletion of miRNA genes or dysregulation of epigenetics [9]. Moreover, miRNAs expression is dysregulated in cancer as an effect of defects in miRNA biogenesis machinery, including DICER and DROSHA [11].

MiRNAs take part in all steps of tumor cell invasiveness and metastasis including migration, local invasion, epithelial–mesenchymal transition (EMT), and systemic circulation [12]. The same miRNAs may play opposite roles in different tumors, promoting tumor growth (oncomiRNAs) or acting as tumor suppressor miRNAs [13]. By targeting 3′ untranslated region (UTR) of multiple mRNAs they regulate all hallmarks of cancer defined by Hanahan and Weinberg, including proliferation, invasion, angiogenesis, as well as they influence cancer cells chemoresistance [12,14,15,16,17].

This systematic review aims to highlight the complex role of miRNAs in regulating endometrial cancer invasion and metastasis. We focus on the aberrant expression of different miRNAs in endometrial cancer tissues and cell lines and their role in the regulation of tumor invasion, metastasis, and patients’ outcomes.

## 2. Materials and Methods

### 2.1. Search Strategy

**Figure 2:**
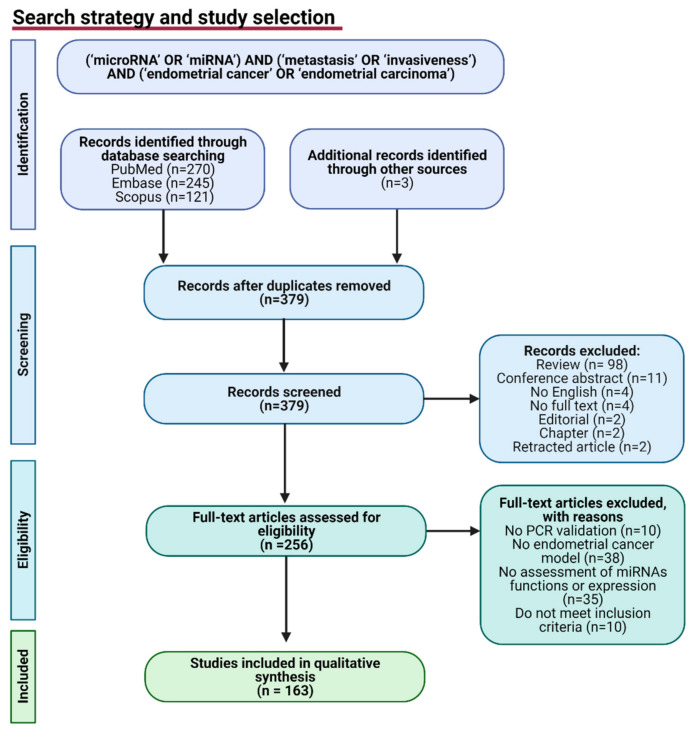
PRISMA flow-chart diagram of study selection. n = number of articles. Figure was created using Biorender.com.

The Preferred Reporting Items for Systematic Reviews and Meta-Analyses (PRISMA) were used to ensure reporting transparency (Figure 2). Two reviewers (KK and TMG) collected, screened, and performed an independent assessment of the quality of the studies. Discrepancies were discussed and resolved by consensus. The literature systematic search was undertaken using MEDLINE (PubMed), Embase, and Scopus (9 March 2021) with the terms (‘microRNA’ OR ‘miRNA’) AND (‘metastasis’ OR ‘invasiveness’) AND (‘endometrial cancer’ OR ‘endometrial carcinoma’). Duplicates were deleted. The references of the found studies were reviewed to find other records.

### 2.2. Inclusion and Exclusion Criteria

The articles were included if they answered the PICO question (Table 1). The studies with the assessment of miRNA expression levels and the role of miRNAs in endometrial cancer cell invasion, migration, or outcome were allowed. We assessed only original articles written in English with full-text available. Review articles, chapters, conference abstracts, and retracted articles were excluded. Full texts were assessed for eligibility. We excluded articles that did not meet inclusion criteria, articles with no assessment of miRNAs function or expression in the endometrial cancer model, and no validation with RT-qPCR (real-time quantitative PCR) of miRNA expression.

### 2.3. Data Extraction

The data extracted from the revised full-texts were the levels of expression of miRNAs in human tissues, human serum, and cell lines, the role of miRNAs in the regulation of migration, invasion, and EMT in vitro, as well as tumor growth and metastasis in vivo, the outcomes of patients, the overall survival (OS), disease-free survival (DFS), progression-free survival (PFS), FIGO stage, histological grade, myometrial invasion, and lymph node metastases.

## 3. Results

The detailed results of the literature search are presented in Figure 2. The search strategy provided a total of 270 records in the PubMed database, 245 in the Embase, 121 in the Scopus database, and three records identified through other sources. In total, 379 records remained after the removal of duplicated articles. Out of 379, 123 records were excluded with reasons (no validation by RT-qPCR, no EC model, no assessment of miRNA functions or expression, or records that do not meet inclusion criteria). A total of 256 full-text articles were assessed for eligibility. Finally, data were collected from 163 articles demonstrating the role of miRNA in the regulation of EC invasiveness or metastasis.

### 3.1. MiRNAs Dysregulation in Endometrial Cancer

Our search identified 115 articles that revealed 106 miRNAs regulating invasiveness and metastasis that are dysregulated in EC, which was confirmed by RT-qPCR (Table 2) [18,19,20,21,22,23,24,25,26,27,28,29,30,31,32,33,34,35,36,37,38,39,40,41,42,43,44,45,46,47,48,49,50,51,52,53,54,55,56,57,58,59,60,61,62,63,64,65,66,67,68,69,70,71,72,73,74,75,76,77,78,79,80,81,82,83,84,85,86,87,88,89,90,91,92,93,94,95,96,97,98,99,100,101,102,103,104,105,106,107,108,109,110,111,112,113,114,115,116,117,118,119,120,121,122,123,124,125,126,127,128,129,130,131,132]. In total, 33 miRNAs were confirmed to be dysregulated by at least two studies. Most of them (63) are tumor suppressors and are downregulated in EC, while 38 are oncomiRNAs upregulated in cancer tissue. Five miRNAs have discordant expression levels in different studies.

### 3.2. MiRNAs Regulating Endometrial Cancer (EC) Invasiveness and Metastasis

**Figure 3:**
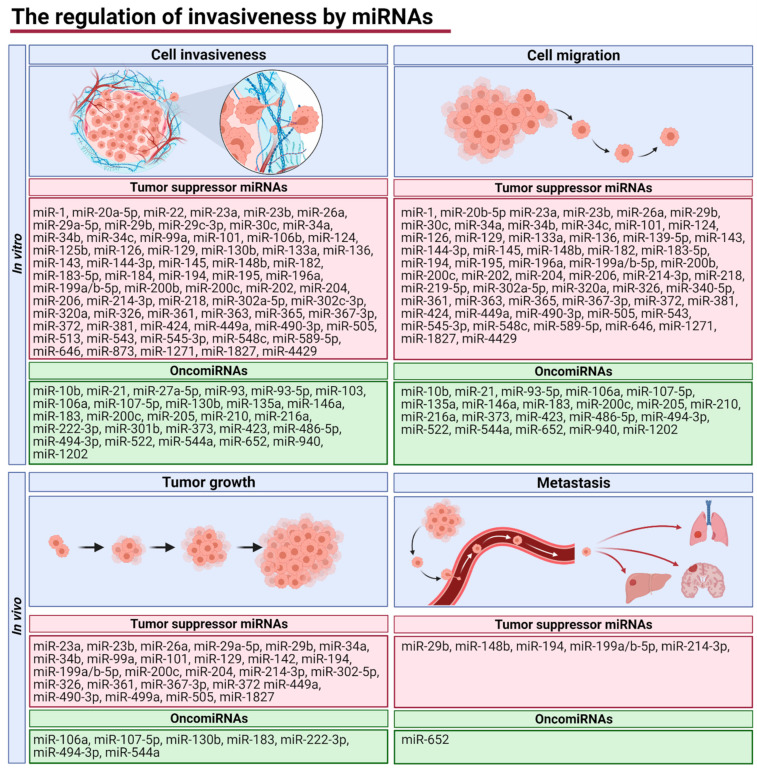
miRNAs regulating cell invasiveness and cell migration in vitro as well as tumor growth, and metastasis in vivo. Figure was created using Biorender.com.

Further, we collected data from articles investigating the role of miRNAs in the regulation of EC invasiveness and metastasis. We identified 132 articles demonstrating the role of 97 miRNAs in the regulation of migration, invasiveness, and EMT of endometrial cancer cell lines in vitro [18,19,20,21,22,23,24,25,26,27,28,30,31,32,33,34,36,39,40,41,42,43,44,45,46,49,50,51,52,53,54,55,56,58,62,63,64,65,66,67,68,69,70,71,72,73,74,79,80,82,83,85,86,87,88,91,92,93,94,95,96,97,98,100,101,102,103,105,106,107,108,109,111,112,113,114,115,116,117,119,121,122,123,124,125,126,127,128,129,130,131,133,134,135,136,137,138,139,140,141,142,143,144,145,146,147,148,149,150,151,152,153,154,155,156,157,158,159,160,161,162,163,164,165,166,167,168,169,170,171,172,173]. From them, 69 miRNAs act as tumor-suppressor miRNAs and 26 are oncomiRNAs, and two miRNAs (miR-130b and miR-200c) have an unclear role due to inconsistent data (Figure 3).

Included studies involved established EC cell lines, including Ishikawa, HEC-1A, HEC-1B, HHUA, AN3CA, ECC-1, RL-95-2, KLE, SPAC-1-L, HEC-50, HOUA-I, and JEC cell line (EC cell lines have been reviewed by Van Nyen et al. [174]). Transient upregulation of a given miRNA with synthetic miRNA or downregulation with complementary anti-miRNA revealed regulation of tumor cell migration and invasiveness in vitro assays by 97 miRNAs. Further, luciferase reporter assays confirmed direct binding of 102 targets by miRNAs regulating invasiveness of EC cells creating a complex regulatory network. Tumor-suppressor miRNAs that are downregulated in EC were identified to bind 77 targets, including some of the well-known oncogenes. OncomiRNAs were identified to promote EC cell migration and invasiveness by binding 25 targets.

Moreover, 35 studies investigated the role of miRNAs in the regulation of tumor growth and EC metastasis in vivo in murine models [18,19,24,36,41,42,44,45,49,51,52,53,56,62,69,70,72,73,92,93,101,111,117,119,122,126,128,136,137,144,145,151,157,160,166]. Eight miRNAs were identified as oncomiRNAs in vivo (miR-106a, miR-107-5p, miR-130b, miR-183, miR-222-3p, miR-494-3p, miR-544a, and miR-652) and 27 as tumor suppressor miRNAs (miR-23a, miR-23b, miR-26a, miR-29a-5p, miR-29b, miR-34a, miR-34b, miR-99a, miR-101, miR-129, miR-142, miR-148b, miR-194, miR-199a/b-5p, miR-200c, miR-204, miR-214-3p, miR-302a-5p, miR-326, miR-361, miR-367-3p, miR-372, miR-449a, miR-490-3p, miR-499a, miR-505, and miR-1827). All but miR-148b and miR-652 were found to regulate primary tumor growth. Moreover, miR-29b, miR-148b, miR-194, miR-199a/b-5p and miR-214-3p were identified to suppress tumor metastasis while miR-652 were found to promote this process. Most of the studies used xenograft assays with human HEC-1B or Ishikawa EC cell lines in immunocompromised mice (Table 3).

**Figure 4:**
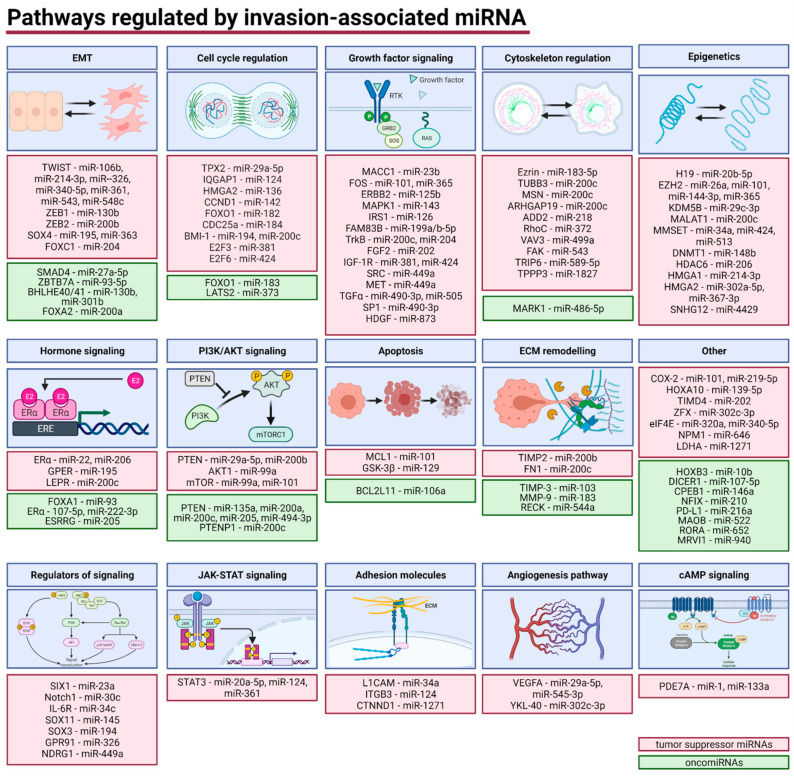
Direct targets of invasion-regulating miRNAs. cAMP—Cyclic Adenosine Monophosphate, EMT—epithelial–mesenchymal transition, ECM—extracellular matrix, JAK-STAT—Janus Kinase/Signal Transducers and Activators of Transcription, PI3K/AKT—Phosphatidylinositol 3-Kinase/Protein Kinase B. Figure was created using Biorender.com.

We categorized miRNAs’ targets into 15 groups based on their biological function in cancer (Figure 4). Tumor suppressor miRNAs that inhibit cell invasiveness and migration and were downregulated in EC (Table 2) were found to target regulators of EMT (5 targets, 12 miRNAs), growth factor signaling (14 targets, 18 miRNAs), cell cycle (9 targets, 10 miRNAs), cytoskeleton (10 targets, 10 miRNAs), hormone signaling (3 targets, 4 miRNAs), phosphatidylinositol 3-kinase/protein kinase B (PI3K/AKT) signaling (3 targets, 5 miRNAs), epigenetic regulators (10 targets, 16 miRNAs). Moreover, tumor suppressor miRNAs were identified to target regulators of signaling pathways (7 targets, 7 miRNAs), extracellular matrix (ECM) remodeling (2 targets, 2 miRNAs), adhesion molecules (3 targets, 3 miRNAs), angiogenesis pathway (2 targets, 3 miRNAs), Janus kinase/signal transducers and activators of transcription (JAK-STAT) signaling pathway (1 target, 3 miRNAs), apoptosis (2 targets, 2 miRNAs), cyclic adenosine monophosphate (cAMP) signaling (1 target, 2 miRNAs), and various other mRNAs (7 targets, 9 miRNAs). On the contrary, oncomiRNAs that were upregulated in EC target regulators of EMT (4 targets, 5 miRNAs), hormone signaling (3 targets, 4 miRNAs), ECM remodeling (3 targets, 3 miRNAs), PI3K/AKT signaling (2 targets, 6 miRNAs), cell cycle (2 targets, 2 miRNAs), cytoskeleton regulators (1 target, 1 miRNA), apoptosis (1 target, 1 miRNA), and others (8 targets, 8 miRNAs).

### 3.3. Relationship between miRNA Expression and Clinical Parameters

Further, we collected data from articles investigating the association between the expression of invasion-related miRNAs in tumor tissue and clinical parameters, including OS, DFS, PFS, FIGO stage, histological grade, myometrial invasion, and lymph node metastases. We identified 51 articles that correlated upregulated expression of 25 miRNAs and downregulated expression of 41 miRNAs with at least one clinical parameter (Table 4) [18,19,24,25,28,29,32,34,37,38,41,44,49,51,52,56,57,58,60,69,70,71,76,77,78,79,81,84,95,99,100,108,110,111,115,117,120,122,130,131,132,147,154,160,175,176,177,178,179,180,181].

Increased levels of nine miRNAs, miR-93-5p, miR-95, miR-205, miR-301, miR-373, miR-494-3p, miR-522, miR-544a and miR-940 in tumor tissue as well as decreased levels of 22 miRNAs, miR-10b, miR-29b, miR-29c, miR-34a, miR-100, miR-101, miR-124, miR-139-5p, miR-141, miR-142, miR-152, miR-184, miR-194, miR-202, miR-203, miR-301b, miR-424, miR-449a, miR-455-5p, miR-497, miR-513, and miR-548c were correlated with a shorter OS (Table 4). On the contrary, upregulated expression of miR-130b and miR-199a was associated with a longer OS in ECs. Upregulation of four miRNAs, miR-107-5p, miR-130b, miR-301, miR-373 as well as downregulation of eight miRNAs, miR-29a-5p, miR-34a, miR-101, miR-124, miR-125b, miR-142, miR-381, miR-490-3p, were associated with the invasion of the myometrium. We identified nine upregulated miRNAs and 17 downregulated miRNAs that correlated with lymph node metastases. Moreover, an increase of miR-30c-3p, miR-192, miR-194, miR-203, miR-345 and a decrease of miR-195 were associated with lymphovascular invasion (Table 4).

## 4. Discussion

Tumor expansion and progression are enabled by coordinated dysregulation of various mechanisms. The process of tumor invasion and metastasis is composed of several steps, including primary tumor growth, migration, local invasion, intravasation, survival in the circulation, extravasation, and pre-metastatic niche formation. All of these steps are regulated by a variety of different miRNAs [12].

In this work, we provide a comprehensive overview of miRNAs that are dysregulated in EC and contribute to tumor progression. We identified 106 dysregulated miRNAs through a systematic literature review. Small RNA-seq analysis revealed that 239 out of 359 detected miRNAs are dysregulated in EC compared to healthy adjacent endometrial tissue [182]. Further, analysis of miRNA-seq data from The Cancer Genome Atlas (TCGA) database expanded the list of dysregulated miRNAs to 531 [179]. Previous systematic reviews identified, respectively, 106 miRNAs [183], 261 miRNAs [184], 310 miRNAs [185] dysregulated in ECs. However, these studies included also many miRNAs detected by high throughput methods, including microarrays and small RNA-seq, without confirmation by the RT-qPCR method that is a gold standard for miRNAs analysis [186]. Moreover, in contrast to previous reviews, we included only miRNAs with a confirmed role in the regulation of EC cell migration, invasiveness, and metastasis in vitro and/or in vivo.

### 4.1. Regulatory Network of Invasion-Associated miRNAs

MiRNAs orchestrate tumor invasion and metastasis by targeting various mRNAs. Complex regulation of multiple signaling pathways, including PI3K/AKT [187], and cellular processes, including EMT and cytoskeleton remodeling [188], by miRNAs enable the control of the invasiveness and metastasis of cancer cells. We classified targets of miRNAs into 15 categories based on their function. Notably, multiple miRNAs target regulators of multiple cellular processes and create a complex network of interactions. Hence, they have been assigned to a given category based on their best-described function in the EC (Figure 4).

#### 4.1.1. Epithelial–Mesenchymal Transition (EMT)

One of the most important processes in the myometrial invasion of EC is an EMT [188]. EMT enables the acquisition of the mesenchymal-like features from the epithelial cells and occurs physiologically during embryonic development and tissue regeneration [189]. EMT is characterized by the loss of adherent junctions, downregulation of epithelial markers (cytokeratins and E-cadherin) but upregulation of mesenchymal markers, including N-cadherin, vimentin, and fibronectin [12,189]. EMT is regulated by multiple signaling pathways, including Transforming Growth Factor β (TGF-β)/SMAD signaling, Wnt pathway, and PI3K/AKT, and is orchestrated by Snail, Slug, Smug, SRY-Box Transcription Factor 1 (SOX1), Forkhead Box C1 (FOXC1), Zinc Finger E-Box Binding Homeobox 1/Zinc Finger E-Box Binding Homeobox 2 (ZEB1/ZEB2), and Twist-related protein 1/Twist-related protein 2 (TWIST1/TWIST2) transcription factors [190,191].

We identified 20 suppressors of this process in EC cells (miR-20a-5p, miR-26a, miR-34a, miR-101, miR-106b, miR-124, miR-130b, miR-183-5p, miR-194, miR-195, miR-199a/b-5p, miR-200c, miR-202, miR-214-3p, miR-320a, miR-326, miR-340-5p, miR-365, miR-424, and miR-513) and seven oncomiRNAs that stimulate cells transition (miR-93, miR-130b, miR-183, miR-200a, miR-200c, miR-205, and miR-301b) (Table 3). Moreover, we identified microRNAs that target nine regulators of EMT, including TWIST, ZEB1/ZEB2, SRY-Box Transcription Factor 4 (SOX4), FOXC1, SMAD4, Zinc Finger and BTB Domain Containing 7A (ZBTB7A), Basic helix-loop-helix transcription factors e40/41 (BHLHE40/41), and Forkhead Box A2 (FOXA2). TWIST, a crucial transcription factor regulating EMT, was identified as a target of several miRNAs in this study (miR-106b, miR-214-3p, miR-326, miR-340-5p, miR-361, miR-543, and miR-548c). Similarly, ZEB1 and ZEB2 transcription factors were identified to be targets of miR-130b and miR-200b, respectively (Figure 4).

#### 4.1.2. Cell Cycle

The cell cycle is regulated by several protein checkpoints including cyclins, cyclin-dependent kinases (CDKs), and their inhibitors (CKI), that may be inactivated or mutated in cancer cells that lead to uncontrolled proliferation and thus to the progression of cancer [192,193]. There are several I-III phases clinical trials of the application of CKI in EC [194]. MiRNAs are described to control the expression of genes related to the cell cycle [195]. In this systematic review, we summarize tumor suppressor miRNAs (miR-29a-5p, miR-124, miR-136, miR-142, miR-182, miR-184, miR-194, miR-200c, miR-381, miR-424) and their targets (TPX2 Microtubule Nucleation Factor (TPX2), IQ Motif Containing GTPase Activating Protein 1 (IQGAP1), High Mobility Group AT-Hook 2 (HMGA2), C-terminal Cyclin D1 (CCND1), Forkhead Box O1 (FOXO1), Cell division cycle 25a (CDC25a), B-lymphoma Moloney murine leukemia virus insertion region-1 (BMI-1), Transcription Factor 3 (E2F3), and E2F Transcription Factor 6 (E2F6)) and oncomiRNAs (miR-183 and miR-373) and their targets (FOXO1 and Large Tumor Suppressor Kinase 2 (LATS2)) involved in cell-cycle regulation. HMGA2 is an oncogene upregulated in several cancers that stimulates proliferation and invasion. HMGA2 is targeted in ECs by tumor suppressor miR-136 [65]. CCND1 activates cyclin-dependent kinase (CDK)4/6 and thus stimulates proliferation, migration, and invasion of cells [196]. In this study, CCND1 was found to be targeted by miR-142 [69].

#### 4.1.3. Growth Factors and Regulators of Signaling

Overactivation of growth factors signaling is crucial in tumor development and progression. Thus, inhibitors of signaling pathways are currently under investigation in EC patients [197]. In this study, we identified multiple growth factors and regulators of signaling that promote EC cell invasion and were targets of different miRNAs. MiRNAs (miR-23b, miR-101, miR-125b, miR-126, miR-143, miR-199a/b-5p, miR-200c, miR-202, miR-204, miR-365, miR-381, miR-424, miR-449a, miR-490-3p, miR-505, and miR-873) that target growth factors (Metastasis-associated in colon cancer protein 1 (MACC1), Fos Proto-Oncogene (FOS), Erb-B2 Receptor Tyrosine Kinase 2 (ERBB2), Mitogen-Activated Protein Kinase (MAPK1), Insulin Receptor Substrate 1 (IRS1), Family With Sequence Similarity 83 Member B (FAM83B), Tropomyosin receptor kinase B (TrkB), Fibroblast growth factor 2 (FGF2), Insulin-like growth factor 1 (IGF-1R), SRC Proto-Oncogene (SRC), MET Proto-Oncogene (MET), Transforming Growth Factor Alpha (TGFα), Sp1 Transcription Factor (SP1), and Hepatoma-derived Growth Factor (HDGF)) decrease migration, invasion, and EMT in vitro and tumor growth and metastases in vivo, as well as correlate with EC stage, grade, and patients outcome. TGFα promotes the progression of cancer by increasing proliferation and differentiation. In our study, miR-490-3p and miR-505 target TGFα and act as tumor suppressor miRNAs [27,70].

Additionally, several tumor-suppressing miRNAs (miR-23a, miR-30c, miR-34c, miR-145, miR-194, miR-326, and miR-449a) target regulators of signaling (SIX Homeobox 1 (SIX1), Notch homolog 1 (Notch1), Interleukin 6 Receptor (IL-6R), SRY-Box Transcription Factor 11 (SOX11), SRY-Box Transcription Factor 3 (SOX3), G protein-coupled receptor 91 (GPR91), and N-Myc Downstream Regulated 1 (NDRG1)). Notch1 is associated with EMT, metastases, and poor prognosis in different cancers including EC [198,199]. MiR-30c targets Notch1 and decreases migration and invasion, however, it is associated with increased lymphovascular invasion in EC patients [96,164,178].

#### 4.1.4. Cytoskeleton Regulation

The polarization of the cytoskeleton and formation of the leading protrusion initiate cell migration [200]. During this process, the cell cytoskeleton undergoes dynamic changes that are regulated by multiple factors interacting with actin microfilaments or tubulin [201]. We identified eight tumor suppressor miRNAs (miR-183-5p, miR-200c, miR-218, miR-372, miR-499a, miR-543, miR-589-5p, and miR-1827) that target regulators of the cytoskeleton (Ezrin, Tubulin Beta 3 Class III (TUBB3), moesin (MSN), Rho GTPase activating protein 19 (ARHGAP19), adducin 2 (ADD2), Ras Homolog Family Member C (RhoC), Vav Guanine Nucleotide Exchange Factor 3 (VAV3), Focal adhesion kinase (FAK), Thyroid Hormone Receptor Interactor 6 (TRIP6), Tubulin Polymerization Promoting Protein Family Member 3 (TPPP3)) and inhibits EC cell migration and invasiveness. Ezrin, which overexpression in EC is related to poor prognosis [202], is targeted by downregulated miR-183-5p. Moreover, a well-described tumor suppressor miR-200c [203] targets TUBB3, MSN, and ARHGAP19, which regulate multiple cytoskeletal-related events. On the contrary, miR-486-5p was found to promote EC cell invasiveness by targeting Microtubule affinity regulating kinase 1 (MARK1) [112], a tumor suppressor in several types of cancer [112,204,205].

#### 4.1.5. Epigenetics

Gene expression is modified by multiple epigenetic mechanisms, including DNA methylation, histone acetylation and methylation, and non-coding RNAs. Dysregulation of these mechanisms in cancer affects a variety of cellular responses [206]. We selected 16 tumor suppressor miRNAs (miR-20b, miR-26a, miR-29c-3p, miR-34a, miR-101, miR-144-3p, miR-148b, miR-200c, miR-206, miR-214-3p, miR-302a-5p, miR-365, miR-367-3p, miR-424, miR-513, miR-4429) that targeted 10 regulators, including Enhancer of Zeste Homolog 2 (EZH2), Lysine Demethylase 5B (KDM5B), Multiple Myeloma SET Domain (MMSET), Metastasis Associated Lung Adenocarcinoma Transcript 1 (MALAT1), DNA methyltransferase 1 (DNMT1), Histone Deacetylase 6 (HDAC6), High Mobility Group AT-Hook 1 (HMGA1), High Mobility Group AT-Hook 2 (HMGA2), Small Nucleolar RNA Host Gene 12 (SNHG12), and H19 Imprinted Maternally Expressed Transcript (H19). EZH2 is the enzymatic catalytic subunit of the polycomb-repressive complex 2 (PRC2) that represses transcription of multiple genes, including tumor-suppressors [207]. It is overexpressed in many types of cancer, including EC [208], and is correlated with decreased DFS and OS [209]. EZH2 is targeted by miR-26a and miR-101, which are downregulated in EC [42,46].

Importantly, epigenetic alterations affect miRNAs profile in EC [185]. It was found that loci of genes coding oncomiRNAs miR-130a/b, miR-182, miR-200b, miR-208a, miR-222, miR-625 are hypo-methylated in EC while genes of tumor-suppressor miRNAs miR-34b, miR-124a-1, miR-124a-2, miR-124a-3, miR-129-2, miR-137, miR-152, miR-638, miR-663 are hyper-methylated [185].

#### 4.1.6. Hormone Signaling

Dysregulation of hormone signaling is one of the most important factors in EC development. From them, estrogen seems to be crucial in EC pathogenesis. Estrogen binds to estrogen receptors (ER) or G protein-coupled estrogen receptor 1 (GPER1) to regulate gene transcription and to promote cancer cell pathways [210,211,212]. Estrogen signaling and miRNAs are in complex interaction since estrogen modulates miRNAs expression but also miRNAs target their receptors [213]. In this study, we identified miRNAs that suppress EC progression via binding to estrogen receptors. MiR-22 and miR-206 target ER what inhibits EC cell invasion and migration [121,156,168]. Moreover, miR-195 targets GPER1 and inhibits EC migration, invasion, and EMT in vitro [139]. On the other hand, miR-107-5p and miR-222-3p targeting ERa and miR-205 targeting Estrogen Related Receptor Gamma (ESRRG) act as oncomiRNAs by enhancing migration, invasion in vitro or/and tumor growth in vivo [19,52,68].

#### 4.1.7. Phosphatidylinositol 3-Kinase/Protein Kinase B (PI3K/AKT) Pathways

Estrogen stimulates the PI3K/Akt pathway, one of the most important pathways responsible for EC proliferation, migration, invasion, and EMT [210]. The activation of this oncogenic pathway is regulated by PTEN, a well-known tumor suppressor potently downregulated in EC [214]. Our study highlights oncomiRNAs (miR-135a, miR-200a, miR-200c, miR-205, and miR-494-3p) and tumor suppressor miRNAs (miR-29a-5p, miR-99a, miR-101, and miR-200b) that target members of this pathway.

#### 4.1.8. Apoptosis

Apoptosis is a process of programmed cell death via the activation of different pathways [215]. Novel therapies targeting apoptosis inhibit B-cell lymphoma-2 (Bcl-2), induced Myeloid Leukemia Cell Differentiation Protein 1 (MCL-1), or target the P53 pathway [216]. In this study, we identified tumor suppressor miRs and oncomiRNAs that target mRNAs of proteins involved in the regulation of apoptosis. Tumor suppressor miR-101 that targets MCL-1, a protein belonging to the Bcl-2 family that inhibits apoptosis, inhibits migration, invasion, and EMT in vitro and tumor growth in vivo, and influences patient survival. Moreover, we recognized invasion-suppressing miR-129 that targets Glycogen synthase kinase-3β (GSK3β), a serine/threonine-protein kinase that inhibits apoptosis via NF-κB activation [217]. MiR-106a targets proapoptotic Bcl-2-like protein 11 (BCL2L11) and acts as oncomiRNA and stimulates migration, invasion in vitro, and tumor growth in vivo [218].

#### 4.1.9. Extracellular Matrix (ECM) Remodeling

Remodeling of ECM is crucial for a cancer cell to initiate an invasion of adjacent tissues. Among multiple factors regulating this process, the most important are Matrix Metalloproteinases (MMPs) and their inhibitors Tissue Inhibitors of Metalloproteinases (TIMPs). MMPs degrade collagen, fibrinogen, fibronectin (FN1), and others and in this way enable tumor cells to invade [12]. In this study, miR-200b targets TIMP-2, miR-200c targets FN1 and both miRNAs act as tumor suppressor miRNAs. On the other hand, miR-103 targets TIMP-3 and stimulates invasion. Moreover, oncomiRNA miR-183 targets MMP-9 and miR-544a targets Reversion Inducing Cysteine Rich Protein with Kazal Motifs (RECK). These results show the complexity of the regulatory processes involved in ECM remodeling. One target may be regulated by several miRNAs, as well as miRNAs may have a role in the regulation of invasion by different mRNAs [219].

#### 4.1.10. Janus Kinase/Signal Transducers and Activators of Transcription (JAK-STAT) Signaling

JAK-STAT pathway is a known regulator of tumor progression [220]. JAK-STAT pathway is dysregulated in endometrial cancer cells what leads to increased proliferation [221]. In this study, we identified three miRNAs (miR-20a-5p, miR-124, and miR-361) that target STAT3. MiR-20a-5p inhibits invasion and EMT [40]. MiR-124 decreases migration, invasion, and EMT in vitro and is associated with patients’ outcomes [50,99]. Conversely, miR-361 inhibits migration and invasion in vitro and tumor growth in vivo, as well as is negatively correlated with histological grade [169].

#### 4.1.11. Adhesion Molecules

Tumor cell migration and invasiveness strictly depend on the cell adhesion molecules, which include cadherins, integrins, selectins, Ig-superfamily Cell Adhesion Molecules (CAMs), and others [12,222,223]. We identified three tumor suppressor miRNAs (miR-34a, miR-124, and miR-1271) that target three adhesion molecules (L1 Cell Adhesion Molecule (L1CAM), Integrin Subunit Beta 3 (ITGB3), Catenin Delta 1 (CTNND1)). L1CAM is primarily a regulator of nervous system development [224]. However, further studies demonstrated its role in the regulation of tumor progression [225]. In EC patients, L1CAM is an important prognostic factor and an independent predictor of poor survival [226,227,228]. Upregulation of L1CAM in EC is at least in part caused by the downregulation of L1CAM-targeting tumor suppressor miR-34a.

#### 4.1.12. Angiogenesis

Angiogenesis is one of the hallmarks of cancer according to Hanahan and Weinberg [14] playing a crucial role in the progression of EC. One of the most important proangiogenic factors is the Vascular Endothelial Growth Factor (VEGF) [229]. VEGF is often targeted in novel therapies including anti-VEGF antibody (bevacizumab), VEGF trap (aflibercept), or Tyrosine Kinase Inhibitors (TKI). Therapy with bevacizumab was approved by FDA in different tumors including colorectal cancer and is currently tested in EC [229,230]. An increased number of clinical trials are assessing the efficacy of that novel therapies in monotherapy as well as in a combination with other applied therapies but still lacks a personalized approach [229]. In this study, we found two miRNAs (miR-29a-5p and miR-545-3p) that target VEGF and thus influence EC progression.

#### 4.1.13. Cyclic Adenosine Monophosphate (cAMP) Signaling

cAMP pathways are described to inhibit the invasion of cancer [231,232]. cAMP promotes apoptosis and decreases tumor growth [233]. The cAMP-specific phosphodiesterase 7A (PDE7A) hydrolyzes cAMP [97]. MiR-1 and miR-133a target PDE7A which leads to the inhibition of EC cell migration and invasion in vitro.

### 4.2. The Role of miRNAs in EC Diagnosis and Management

**Figure 5:**
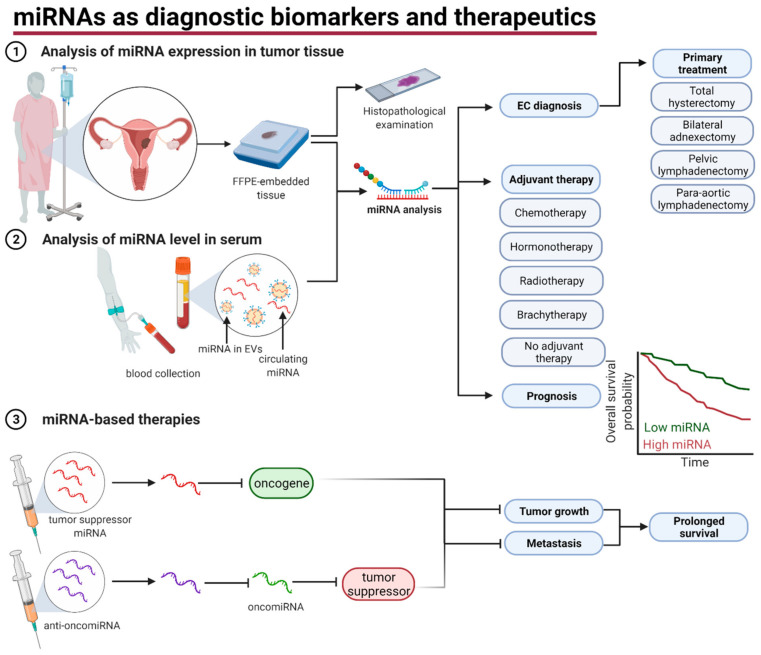
miRNAs as diagnostic biomarkers and therapeutics. miRNAs are promising biomarkers that can be analyzed either from tumor tissue (1) or from blood obtained from EC patients (2). miRNAs may support EC diagnosis and indicate primary and adjuvant treatment. Moreover, their expression is an important prognostic factor. miRNA-based therapies that include tumor suppressor miRNAs and anti-oncomiRNAs are currently tested in preclinical and clinical trials (3). EVs—extracellular vesicles, FFPE—formalin-fixed, paraffin-embedded. Figure was created using Biorender.com.

There is a great clinical interest in the determination of biomarkers for the diagnosis and management of EC, especially to enable individualized cancer care in the light of genomic classification [234]. Despite the identification of many prognostic biomarkers in EC [235,236], none of them are routinely used for diagnostic or prognostic purposes. Recent systematic reviews and meta-analyses revealed the clinical utility for the use of miRNAs as biomarkers in a variety of cancer, including bladder cancer [237], prostate cancer [238], ovarian cancer [239], and breast cancer [240], which makes miRNAs promising candidate for biomarkers in EC (Figure 5).

Importantly, dysregulation of miRNAs expression can be also detected in plasma or serum (Figure 5). It was identified that the concentration of 19 miRNAs is increased in EC patients and the concentration of 10 miRNAs is decreased compared to healthy women [184]. Exploring publications we found that the concentration of six miRNAs was decreased and the concentration of 11 miRNAs was higher in EC patients and five from them (miR-27, miR-29b, miR-95, miR-203, and miR-449a) were associated with tumor myometrial invasion or lymph node metastases [60,77,81,110].

MiRNAs are also promising therapeutics in clinical oncology. Among described in this study miRNAs regulating tumor invasiveness, the effectiveness and safety of miR-34 have been tested in clinical trials. Despite the good efficacy of MRX34 (the liposome-miR-34 complex) in intravenous administration with dexamethasone premedication, the clinical trial was terminated due to severe adverse events including deaths [241,242]. One of the challenges with using miRNAs or their inhibitors in clinical practice is finding the correct route of administration because of their toxicity, limited stability, and low penetrance to target cells. Several delivery strategies have been developed including local delivery, viral delivery, lipid-based and polymer-based vectors [243]. So far, despite numerous preclinical studies and clinical trials none of the miRNA-based therapeutics have complied with expectations [243,244,245].

Some limitations should be acknowledged in this systematic review. First, to minimize the rate of false-positive results, we excluded studies that did not confirm the expression of miRNAs by RT-qPCR. Thus, we excluded studies that performed only global miRNA profiling. It may introduce selection bias and cause exclusion of relevant miRNAs as well as limits the number of comparable results. Second, most of the studies did not distinguish types of EC in the analyzed cohort or focus on EC type I which may introduce bias. Indeed, some studies demonstrate the differences in the miRNA profile in different types of EC [246,247]. Third, the included studies present heterogeneity in sample types (fresh frozen or formalin-fixed, paraffin-embedded (FFPE) tissues), types of controls, and heterogeneity of study group. Fourth, a small number of miRNAs were assessed by at least two independent studies. Moreover, most of the studies that assessed the correlation of miRNA level and clinical parameters relied on a small group of patients and many of the included articles did not report relevant statistical parameters. Therefore, a meta-analysis could not be performed. Despite these limitations, our article is the first systematic review that comprehensively discusses data regarding the role of miRNAs in EC invasiveness and metastasis.

## 5. Conclusions

In this systematic review, we comprehensively reviewed miRNAs that are crucial regulators of EC invasiveness and metastasis. Extensive research revealed a complex regulatory network of tumor suppressors and oncomiRNAs that orchestrate tumor progression. Identified miRNAs control EC cell migration, invasion, and EMT in vitro, as well as tumor growth and lymph node metastases in vivo. These miRNAs regulate EC by targeting various members of pathways involved in diverse steps of cancer progression. That makes miRNAs promising candidates for diagnostic and prognostic biomarkers and potential therapeutic targets. Nonetheless, miRNAs that can be useful in clinical practice remain to be identified yet. Further large translational and clinical studies are needed to assess the clinical utility of miRNAs.

## Figures and Tables

**Table 1:** PICO question form.

Domain	Inclusion Criteria
Patients (P)	Patients with endometrial cancer or endometrial cancer cells
Interventions (I)	Differentially expressed miRNAs
Comparators (C)	Non-neoplastic endometrium or cells
Outcomes (O)	Tumor invasiveness or metastasis

**Table 2:** Dysregulation of expression of invasiveness-associated miRNAs in endometrial cancer.

MiRNA	Human Tissue	Cell Line	Circulating miRNA	Ref.	MiRNA	Human Tissue	Cell Line	Circulating miRNA	Ref.
let-7b	↓	↓	n/d	[41]	miR-10b *	↑	n/d	↑	[118,129]
miR-1	↓	↓	n/d	[97]	miR-21	↑	n/d	n/d	[57,75,107]
miR-10b *	↓	n/d	n/d	[38,78]	miR-27	↑	n/d	↑	[60]
miR-20a-5p	↓	↓	n/d	[40]	miR-34a *	↑	n/d	n/d	[61]
miR-20b-5p	↓	↓	n/d	[116]	miR-93	↑	n/d	n/d	[27]
miR-22	↓	↓	n/d	[77,121]	miR-93-5p	↑	↑	n/d	[95,105]
miR-23a	↓	n/d	↓	[118,128]	miR-95	↑	n/d	↑	[110]
miR-26a	n/d	n/d	↓	[42]	miR-99a	n/d	n/d	↑	[76]
miR-29a-5p	↓	↓	n/d	[44]	miR-100	n/d	n/d	↑	[76]
miR-29b	↓	n/d	↓	[24,38,81]	miR-106a	↑	↑	n/d	[73]
miR-30c	↓	n/d	n/d	[96]	miR-107-5p	↑	↑	n/d	[19]
miR-34	↓	n/d	n/d	[57]	miR-130b *	↑	n/d	n/d	[122]
miR-34a *	↓	n/d	n/d	[34]	miR-141	↑	n/d	↑	[35,47,77]
miR-34b	↓	n/d	n/d	[38,39,101]	miR-145	↑	n/d	n/d	[21]
miR-34c	↓	n/d	n/d	[43,123]	miR-146a	↑	n/d	n/d	[114]
miR-99a	↓	n/d	n/d	[51,76]	miR-148b	↑	n/d	n/d	[57]
miR-100	↓	n/d	n/d	[76]	miR-181a	↑	n/d	n/d	[37]
miR-101	↓	↓	n/d	[38,46,56,106,119]	miR-182	↑	n/d	n/d	[30,47,48,77]
miR-124	↓	n/d	n/d	[33,50,99]	miR-183	↑	n/d	n/d	[30,48,62,77,92]
miR-125	↓	n/d	n/d	[57]	miR-192	↑	n/d	n/d	[30]
miR-125b	↓	n/d	n/d	[63,104]	miR-194	↑	n/d	n/d	[30]
miR-126	↓	n/d	n/d	[109,120]	miR-200a	↑	n/d	↑	[35,48,77,102]
miR-130b *	↓	n/d	n/d	[32]	miR-200b	↑	n/d	n/d	[31,77,102]
miR-133a	↓	↓	n/d	[38,97]	miR-200c	↑	↑	n/d	[26,47,48,53,77]
miR-133b	↓	n/d	n/d	[38]	miR-203	↑	n/d	↑	[77]
miR-136	↓	n/d	n/d	[65]	miR-205*	↑	n/d	n/d	[29,30,35,38,47,48,59,68,77,78,89,91]
miR-139-5p	↓	n/d	n/d	[54]	miR-210	↑	n/d	n/d	[100]
miR-142	↓	n/d	n/d	[69]	miR-218 *	↑	n/d	n/d	[30]
miR-143	↓	↓	↓	[22]	miR-222-3p	↑	↑	n/d	[47,52]
miR-144-3p	↓	↓	n/d	[85]	miR-301	↑	n/d	n/d	[57]
miR-152	↓	n/d	n/d	[38]	miR-373	↑	↑	n/d	[49,99]
miR-183-5p	↓	n/d	n/d	[98]	miR-429	↑	n/d	n/d	[77,102]
miR-184	↓	n/d	n/d	[28]	miR-449	↑	n/d	n/d	[30]
miR-195	↓	↓	n/d	[78,108]	miR-449a	n/d	n/d	↑	[77]
miR-196a	↓	n/d	n/d	[23]	miR-486-5p	↑	n/d	↑	[112]
miR-199a-3p	↓	n/d	n/d	[90]	miR-494-3p	↑	n/d	n/d	[117]
miR-199a/b-5p	↓	↓	n/d	[93]	miR-499	↑	n/d	n/d	[78]
miR-202	↓	↓	n/d	[25,94]	miR-522	↑	↑	n/d	[132]
miR-204	↓	n/d	n/d	[18,30]	miR-544a	↑	↑	n/d	[111]
miR-205 *	n/d	n/d	↓	[110,118]	miR-652	↑	n/d	n/d	[72]
miR-206	↓	↓	n/d	[124]	miR-940	↑	↑	n/d	[115]
miR-214-3p	↓	↓	n/d	[36,82]	miR-1202	↑	n/d	n/d	[23]
miR-218 *	↓	↓	n/d	[125]	miR-1228	n/d	n/d	↑	[77]
miR-219-5p	↓	n/d	n/d	[87]					
miR-301b	n/d	n/d	↓	[77]					
miR-302a-5p	↓	n/d	n/d	[126]					
miR-302c-3p	↓	n/d	n/d	[67]					
miR-320a	↓	n/d	n/d	[103]					
miR-326	↓	↓	n/d	[55]					
miR-340-5p	↓	n/d	n/d	[103]					
miR-361	↓	↓	n/d	[41]					
miR-363	↓	↓	n/d	[83]					
miR-365	↓	↓	n/d	[80]					
miR-367-3p	↓	n/d	n/d	[126]					
miR-372	↓	n/d	n/d	[130]					
miR-381	↓	↓	n/d	[79]					
miR-424	↓	↓	n/d	[34,58,66]					
miR-449a	↓	n/d	n/d	[127]					
miR-490-3p	↓	n/d	n/d	[64,70]					
miR-499a	↓	↓	n/d	[45]					
miR-505	↓	n/d	n/d	[131]					
miR-513	↓	n/d	n/d	[34]					
miR-543	↓	↓	n/d	[20]					
miR-548c	↓	n/d	n/d	[71]					
miR-589-5p	↓	n/d	n/d	[86]					
miR-646	↓	↓	n/d	[113]					
miR-873	↓	↓	n/d	[84]					
miR-1271-5p	↓	↓	n/d	[74,88]					

↓—downregulated in endometrial cancer compared to normal cells, ↑—upregulated in endometrial cancer compared to normal cells, n/d—no data, *—inconsistent data of miRNAs expression.

**Table 3:** The role of miRNAs in invasion and metastasis in vitro and in vivo.

MiRNA	Target ^1^	Migration	Invasion	EMT	Tumor Growth In Vivo	Metastasis In Vivo	Ref.
miR-1	PDE7A	↓	↓	n/d	n/d	n/d	[97]
miR-20a-5p	STAT3	n/d	↓	↓	n/d	n/d	[40]
miR-20b-5p	H19	↓	n/d	n/d	n/d	n/d	[116]
miR-22	ERα	n/d	↓	n/d	n/d	n/d	[121,156]
miR-23a	SIX1	↓	↓	n/d	↓	n/d	[128]
miR-23b	MACC1	↓	↓	n/d	↓	n/d	[137]
miR-26a	EZH2	↓	↓	↓	↓	n/d	[42]
miR-29a-5p	TPX2	n/d	↓	n/d	↓	n/d	[44]
miR-29b	VEGFA, PTEN	↓	↓	n/d	↓	↓	[24,150]
miR-29c-3p	KDM5B	n/d	↓	n/d	n/d	n/d	[154]
miR-30c	MTA-1	↓	↓	n/d	n/d	n/d	[96,173]
miR-34a	Notch1, L1CAM, MMSET	↓	↓	↓	↓	n/d	[34,166,170]
miR-34b	n/d	↓	↓	n/d	↓	n/d	[39,101]
miR-34c	IL-6R	↓	↓	n/d	n/d	n/d	[43,123]
miR-99a	AKT1, mTOR	n/d	↓	n/d	↓	n/d	[51]
miR-101	COX-2, EZH2, MCL-1, FOS, mTOR	↓	↓	↓	↓	n/d	[46,56,106,119]
miR-106b	TWIST1	n/d	↓	↓	n/d	n/d	[140]
miR-124	STAT3, IQGAP1, ITGB3	↓	↓	↓	n/d	n/d	[33,50,167]
miR-125b	ERBB2	n/d	↓	n/d	n/d	n/d	[63,161]
miR-126	IRS1	↓	↓	n/d	n/d	n/d	[109]
miR-129	GSK-3β	↓	↓	n/d	↓	n/d	[136]
miR-130b*	ZEB1	n/d	↓	↓	n/d	n/d	[32]
miR-133a	PDE7A	↓	↓	n/d	n/d	n/d	[97]
miR-136	HMGA2	↓	↓	n/d	n/d	n/d	[65]
miR-139-5p	HOXA10	↓	n/d	n/d	n/d	n/d	[54]
miR-142	CCND1	n/d	n/d	n/d	↓	n/d	[69]
miR-143	MAPK1	↓	↓	n/d	n/d	n/d	[22]
miR-144-3p	EZH2	↓	↓	n/d	n/d	n/d	[85]
miR-145	SOX11	↓	↓	n/d	n/d	n/d	[21]
miR-148b	DNMT1	↓	↓	n/d	n/d	↓	[151]
miR-182	FOXO1	↓	↓	n/d	n/d	n/d	[142,163]
miR-183-5p	Ezrin	↓	↓	↓	n/d	n/d	[98]
miR-184	CDC25A	n/d	↓	n/d	n/d	n/d	[28]
miR-194	BMI-1, Sox3	↓	↓	↓	↓	↓	[141,145]
miR-195	SOX4, GPER	↓	↓	↓	n/d	n/d	[108,139]
miR-196a	n/d	↓	↓	n/d	n/d	n/d	[23]
miR-199a/b-5p	FAM83B	↓	↓	↓	↓	↓	[93]
miR-200b	TIMP2, PTEN, ZEB2	↓	↓	n/d	n/d	n/d	[31,102,133]
miR-200c *	TUBB3, BMI-1, MSN, FN1, TrkB, ARHGAP19, LEPR	↓	↓	↓	↓	n/d	[138,146,152]
miR-202	FGF2, TIMD4	↓	↓	↓	n/d	n/d	[25,94]
miR-204	TrkB, FOXC1	↓	↓	n/d	↓	n/d	[18,30]
miR-206	HDAC6, ERα	↓	↓	n/d	n/d	n/d	[124,168]
miR-214-3p	TWIST1, HMGA1	↓	↓	↓	↓	↓	[36,82]
miR-218	ADD2	↓	↓	n/d	n/d	n/d	[125]
miR-219-5p	COX-2	↓	n/d	n/d	n/d	n/d	[87]
miR-302a-5p	HMGA2	↓	↓	n/d	↓	n/d	[126]
miR-302c-3p	ZFX, YKL-40	n/d	↓	n/d	n/d	n/d	[67]
miR-320a	eIF4E	↓	↓	↓	n/d	n/d	[103]
miR-326	TWIST1, GPR91	↓	↓	↓	↓	n/d	[55,144]
miR-340-5p	eIF4E	↓	n/d	↓	n/d	n/d	[103]
miR-361	TWIST, STAT3	↓	↓	n/d	↓	n/d	[41,169]
miR-363	SOX4	↓	↓	n/d	n/d	n/d	[83]
miR-365	EZH2, FOS	↓	↓	↓	n/d	n/d	[80]
miR-367-3p	HMGA2	↓	↓	n/d	↓	n/d	[126]
miR-372	RhoC	↓	↓	n/d	↓	n/d	[130]
miR-381	IGF-1R, E2F3	↓	↓	n/d	n/d	n/d	[79,148]
miR-424	IGF-1R, E2F6, MMSET	↓	↓	↓	n/d	n/d	[34,58,66]
miR-449a	NDRG1, SRC, MET	↓	↓	n/d	↓	n/d	[127,143,147,160,172]
miR-490-3p	TGFα, SP1	↓	↓	n/d	↓	n/d	[64,70]
miR-499a	VAV3	n/d	n/d	n/d	↓	n/d	[45]
miR-505	TGFα	↓	↓	n/d	↓	n/d	[131]
miR-513	MMSET	n/d	↓	↓	n/d	n/d	[34]
miR-543	FAK, TWIST1	↓	↓	n/d	n/d	n/d	[20]
miR-545-3p	VEGF	↓	↓	n/d	n/d	n/d	[165]
miR-548c	TWIST	↓	↓	n/d	n/d	n/d	[71]
miR-589-5p	TRIP6	↓	↓	n/d	n/d	n/d	[86]
miR-646	NPM1	↓	↓	n/d	n/d	n/d	[113,155]
miR-873	HDGF	n/d	↓	n/d	n/d	n/d	[84]
miR-1271	LDHA, CTNND1	↓	↓	n/d	n/d	n/d	[74,88]
miR-1827	TPPP3	↓	↓	n/d	↓	n/d	[157]
miR-4429	SNHG12	↓	↓	n/d	n/d	n/d	[134]
miR-10b	HOXB3	↑	↑	n/d	n/d	n/d	[129]
miR-21	n/d	↑	↑	n/d	n/d	n/d	[107]
miR-27a-5p	SMAD4	n/d	↑	n/d	n/d	n/d	[135]
miR-93	FOXA1	n/d	↑	↑	n/d	n/d	[27]
miR-93-5p	ZBTB7A	↑	↑	n/d	n/d	n/d	[95,105]
miR-103	TIMP3	n/d	↑	n/d	n/d	n/d	[164]
miR-106a	BCL2L11	↑	↑	n/d	↑	n/d	[73]
miR-107-5p	ERα	↑	↑	n/d	↑	n/d	[19]
miR-130b *	DICER1, BHLHE40/41	n/d	↑	↑	↑	n/d	[122,171]
miR-135a	PTEN	↑	↑	n/d	n/d	n/d	[159]
miR-146a	n/d	↑	↑	n/d	n/d	n/d	[114]
miR-183	CPEB1, MMP-9, FOXO1	↑	↑	↑	↑	n/d	[62,92,142]
miR-200a	FOXA2, PTEN	n/d	n/d	↑	n/d	n/d	[102,158]
miR-200c *	PTENP1, PTEN, MALAT1	↑	↑	↑	n/d	n/d	[26,53]
miR-205	ESRRG, PTEN	↑	↑	↑	n/d	n/d	[68,91,149]
miR-210	NFIX	↑	↑	n/d	n/d	n/d	[100]
miR-216a	PD-L1	↑	↑	n/d	n/d	n/d	[162]
miR-222-3p	ERa	n/d	↑	n/d	↑	n/d	[52]
miR-301b	BHLHE40/41	n/d	↑	↑	n/d	n/d	[171]
miR-373	LATS2	↑	↑	n/d	n/d	n/d	[49]
miR-423	n/d	↑	↑	n/d	n/d	n/d	[153]
miR-486-5p	MARK1	↑	↑	n/d	n/d	n/d	[112]
miR-494-3p	PTEN	↑	↑	n/d	↑	n/d	[117]
miR-522	MAOB	↑	↑	n/d	n/d	n/d	[132]
miR-544a	RECK	↑	↑	n/d	↑	n/d	[111]
miR-652	RORA	↑	↑	n/d	n/d	↑	[72]
miR-940	MRVI1	↑	↑	n/d	n/d	n/d	[115]
miR-1202	n/d	↑	↑	n/d	n/d	n/d	[23]

^1^—direct binding confirmed by luciferase assay, ↓—downregulated in endometrial cancer compared to normal cells, ↑—upregulated in endometrial cancer compared to normal cells, n/d—no data, EMT—epithelial–mesenchymal transition. *—inconsistent data of miRNAs role.

**Table 4:** The correlation of miRNA expression and clinical parameters.

MiRNA	Expression	OS	DFS or PFS	FIGO Stage	Histological Grade	Myometrial Invasion	Lymph Node Metastases	Ref.
miR-10a	Upregulation	n/d	n/d	n/d	n/d	n/d	↑	[29]
miR-27a	Upregulation	n/d	n/d	↑	n/d	n/d	n/d	[60]
miR-30c-3p	Upregulation	n/d	n/d	n/d	n/d	n/d	↑a	[178]
miR-34a *	Upregulation	n/d	n/d	n/d	n/d	n/d	↑	[29]
miR-93-5p	Upregulation	↓	n/d	↑	n/d	n/d	↑	[95]
miR-95	Upregulation	↓	n/d	↑	↑	n/d	↑	[29,110]
miR-107-5p	Upregulation	n/d	n/d	↑	n/d	↑	↑	[19]
miR-130b	Upregulation	↑	n/d	↑	n/d	↑	n/d	[32,122]
miR-181a	Upregulation	n/d	n/d	↑	n/d	n/d	n/d	[37]
miR-192	Upregulation	n/d	n/d	n/d	n/d	n/d	↑a	[178]
miR-194	Upregulation	n/d	n/d	n/d	n/d	n/d	↑a	[178]
miR-199a	Upregulation	↑	↑	n/d	n/d	n/d	n/d	[177]
miR-200a	Upregulation	n/d	n/d	↑	n/d	n/d	n/d	[29]
miR-203	Upregulation	n/d	n/d	n/d	n/d	n/d	↑a	[178]
miR-205	Upregulation	↓	n/d	↑	n/d	n/d	n/d	[29]
miR-210	Upregulation	n/d	n/d	↑	↑	n/d	↑	[100,178]
miR-222-3p	Upregulation	n/d	n/d	↑	↑	n/d	↑	[52]
miR-301	Upregulation	↓	n/d	↑	↑	↑	↑	[57,178]
miR-345	Upregulation	n/d	n/d	n/d	n/d	n/d	↑a	[178]
miR-373	Upregulation	↓	n/d	↑	↑	↑	↑	[49,99]
miR-494-3p	Upregulation	↓	n/d	n/d	n/d	n/d	n/d	[117]
miR-499	Upregulation	n/d	n/d	↑	↑	n/d	n/d	[78]
miR-522	Upregulation	↓	n/d	n/d	↑	n/d	n/d	[132]
miR-544a	Upregulation	↓	n/d	n/d	n/d	n/d	n/d	[111]
miR-940	Upregulation	↓	n/d	n/d	↑	n/d	n/d	[115]
miR-10b	Downregulation	↓	n/d	n/d	n/d	n/d	n/d	[38]
miR-29b	Downregulation	↓	↓	↑	n/d	n/d	↑	[24,38,81]
miR-29a-5p	Downregulation	n/d	n/d	↑	↑	↑	↑	[44]
miR-29c	Downregulation	↓	n/d	n/d	n/d	n/d	n/d	[154]
miR-34a *	Downregulation	↓	n/d	↑	↑	↑	↑	[34,175]
miR-34b-5p	Downregulation	n/d	n/d	n/d	n/d	n/d	↑	[176]
miR-34c-3p	Downregulation	n/d	n/d	n/d	n/d	n/d	↑	[176]
miR-34c-5p	Downregulation	n/d	n/d	n/d	n/d	n/d	↑	[176]
miR-100	Downregulation	↓	n/d	n/d	n/d	n/d	n/d	[76]
miR-101	Downregulation	↓	n/d	n/d	↑	↑	n/d	[38,56,175]
miR-124	Downregulation	↓	n/d	↑	↑	↑	↑	[99]
miR-125b	Downregulation	n/d	n/d	n/d	↑	↑	n/d	[175]
miR-126	Downregulation	n/d	n/d	↑	↑	n/d	n/d	[120]
miR-139-5p	Downregulation	↓	n/d	n/d	n/d	n/d	n/d	[38]
miR-141	Downregulation	↓	↓	n/d	n/d	n/d	n/d	[77]
miR-142	Downregulation	↓	n/d	n/d	↑	↑	n/d	[69,175]
miR-152	Downregulation	↓	↓	n/d	n/d	n/d	n/d	[38]
miR-184	Downregulation	↓	n/d	n/d	n/d	n/d	↑	[28,176]
miR-194	Downregulation	↓	n/d	↑	n/d	n/d	n/d	[180]
miR-195	Downregulation	n/d	n/d	↑	n/d	n/d	↑a	[108]
miR-202	Downregulation	↓	n/d	↑	n/d	n/d	↑	[25]
miR-203	Downregulation	↓	↓	n/d	n/d	n/d	n/d	[77]
miR-204	Downregulation	n/d	n/d	↑	n/d	n/d	↑	[18]
miR-301b	Downregulation	↓	↓	n/d	n/d	n/d	n/d	[77]
miR-361	Downregulation	n/d	n/d	n/d	↑	n/d	n/d	[41]
miR-372	Downregulation	n/d	n/d	n/d	n/d	n/d	↑	[130]
miR-375	Downregulation	n/d	n/d	n/d	n/d	n/d	↑	[176]
miR-381	Downregulation	n/d	n/d	↑	n/d	↑	↑	[79]
miR-424	Downregulation	↓	n/d	↑	↑	n/d	↑	[34,58]
miR-429	Downregulation	n/d	↓	n/d	n/d	n/d	n/d	[77]
miR-449a	Downregulation	↓	n/d	↑	↑	n/d	↑	[147,160]
miR-455-5p	Downregulation	↓	↓	n/d	n/d	n/d	n/d	[38]
miR-490-3p	Downregulation	n/d	n/d	n/d	n/d	↑	↑	[70]
miR-497	Downregulation	↓	n/d	↑	↑	n/d	n/d	[179]
miR-505	Downregulation	n/d	n/d	↑	n/d	n/d	↑	[131]
miR-513	Downregulation	↓	n/d	n/d	n/d	n/d	n/d	[34]
miR-548c	Downregulation	↓	n/d	n/d	n/d	n/d	n/d	[71]
miR-873	Downregulation	n/d	n/d	↑	↑	n/d	n/d	[84]
miR-99a	Downregulation	n/d	n/d	↑	↑	n/d	n/d	[51]
miR-200 family	Downregulation	n/d	n/d	n/d	↑	n/d	n/d	[181]
miR-1228	Downregulation	n/d	↓	n/d	n/d	n/d	n/d	[77]

↓—decreased, ↑—increased, n/d—no data, a—lympho-vascular invasion, *—inconsistent data of miRNAs expression and clinical parameters, OS—overall survival, DFS—disease-free survival, PFS—progression-free survival, FIGO stage—The International Federation of Gynecology and Obstetrics staging classification.
